# Supplementary material for: Evolutionary shifts in taste coding in the fruit pest Drosophila suzukii
Source: eLife. 2021 Feb 22;10:e64317. doi: 10.7554/eLife.64317 (PMC7899650; doi:10.7554/eLife.64317)
Supplement: Figure 4—source data 1. — (A) Responses in spikes/s of labellar sensilla of three species to bitter compounds. Values represent the mean responses in spikes per second as measured by the number of action potentials generated over a 500 ms interval. For Drosophila suzukii, n = 5–10 for 84% of the 459 tastant-sensillum combinations; n > 10 for the remaining 16%. For Drosophila biarmipes, n = 5–10 for 96% of the 459 tastant-sensillum combinations; n > 10 for the remaining 4%. Responses to the tricholine citrate (TCC) diluent have been subtracted from the tastant responses. (B) SEMs of responses shown in (A). Data from Drosophila melanogaster are from Weiss et al., 2011. [file elife-64317-fig4-data1.pdf]

A

Table. S1

D. suzukii

	L1	L2	L3	L4	L5	L6	L7	L8	L9	I0	I1	I2	I3	I4	I5	I6	I7	I8	S1	S2	S3	S4	S5	S6	S7	S8	S9
TCC	0.0	0.0	0.0	0.0	0.0	0.0	0.0	0.0	0.0	0.0	0.0	0.0	0.0	0.0	0.0	0.0	0.0	0.0	0.8	1.7	0.5	0.0	1.4	13.6	1.9	2.0	0.0
CAF	0.0	0.0	0.0	0.0	0.0	2.0	2.0	0.8	0.0	7.1	5.7	7.5	7.5	8.7	6.0	8.0	8.0	6.3	10.7	7.0	7.6	23.8	6	17.3	4.9	5.7	16.7
UMB	2.8	0.0	3.2	1.6	0.0	0.0	0.0	3.6	1.6	11.5	9.3	11.7	8.7	11.3	11.8	9.7	8.0	7.6	36.1	27.3	-0.2	31.6	28.8	32.7	0.5	27.4	25.3
TPH	0.3	0.0	0.8	0.0	0.0	0.0	0.0	0.0	0.0	6.6	3.0	5.0	5.7	3.7	5.3	1.2	1.7	0.2	19.0	-0.7	0.5	14.1	-0.1	15.7	-1.2	-0.5	12.7
DEN	0.0	0.7	0.3	0.0	0.0	0.0	0.0	0.0	0.0	0.0	0.4	1.7	1.3	0.3	4.7	5.1	3.6	7.2	16.7	-1.7	-0.5	7.4	-1.7	13.4	-1.9	-2.0	11.6
BER	0.0	0.3	0.0	0.0	0.4	0.4	0.0	0.0	0.8	7.3	3.8	6.2	11.6	5.6	8.9	6.6	10.9	10.3	6.3	3.8	9.3	7.9	2.3	19.0	-1.6	-0.7	10.7
LOB	0.4	0.0	0.0	0.0	0.0	0.0	0.0	0.0	0.0	8.0	13.8	12.9	11.1	23.1	22.0	25.3	16.3	9.0	11.6	-1.7	-0.5	11.8	-1.4	22.6	-1.6	-1.1	16.0
SPS	0.0	0.0	0.0	0.0	0.0	0.0	0.0	0.0	0.0	24.0	16.2	19.4	12.7	16.0	16.3	23.6	26.4	29.6	5.9	-1.2	-0.2	1.4	-1.7	45.2	-1.9	-2.0	6.0
ESC	0.0	0.0	0.0	0.0	0.3	0.0	0.0	0.0	0.0	12.3	9.3	8.7	9.6	12.2	10.1	6.9	8.0	10.3	51.2	26.7	2.3	53.5	23.2	39.9	-0.1	22.3	57.6
SAP	0.8	0.0	0.0	2.0	0.0	0.0	0.4	1.6	0.4	0.7	0.0	1.2	0.0	0.8	0.4	2.0	0.4	0.0	2.9	-0.9	-0.5	4.0	3.1	9.7	-1.6	5.6	0.4
AZA	1.6	0.0	0.0	0.4	2.4	0.8	0.0	0.0	1.6	0.0	2.4	2.0	3.6	1.3	0.0	2.0	0.4	0.0	16.0	6.3	-0.5	11.3	0.3	10.0	-0.2	3.6	10.8
COU	0.0	0.0	0.0	0.0	0.0	0.0	0.0	0.0	0.0	0.8	3.6	2.0	4.4	3.2	3.2	2.4	2.4	1.2	20.4	1.0	1.5	19.6	-1.3	26.7	-1.9	1.3	15.4
SOA	0.0	0.0	0.0	0.0	0.0	0.0	0.0	0.0	0.0	0.4	0.0	1.2	0.0	0.0	0.0	0.0	0.0	0.0	18.4	-1.7	-0.5	19	-1.7	24.2	-1.9	-2.0	13.3
DEET	0.0	0.0	0.0	0.0	0.0	0.0	0.0	0.0	0.0	0.0	0.0	0.0	0.3	0.0	0.4	0.0	0.4	0.0	8.2	-1.3	-0.5	3.2	-1.7	-2.8	-1.9	-2.0	3.6
STR	0.0	0.0	0.0	0.0	0.0	0.0	0.0	0.0	0.4	0.0	0.0	0.0	0.4	0.0	0.0	0.0	0.0	0.4	24	-1.7	-0.5	19.4	-1.3	30.8	-1.9	-2.0	11.6
GOS	0.0	0.0	0.0	0.0	0.0	0.0	0.0	0.0	0.0	0.0	0.0	0.3	0.0	0.0	0.0	0.0	0.0	0.0	2.8	17.3	0.2	1.0	20.8	5.9	-1.9	15.6	2.0
ARI	0.0	0.0	0.0	0.0	0.0	0.0	0.0	0.0	0.0	0.8	0.4	0.8	0.0	0.4	0.4	0.0	2.0	0.4	53.2	24.3	9.1	60.0	37.1	39.2	3.0	50.4	53.2

D. biarmipes

	L1	L2	L3	L4	L5	L6	L7	L8	L9	I0	I1	I2	I3	I4	I5	I6	I7	I8	S1	S2	S3	S4	S5	S6	S7	S8	S9
TCC	0.4	6.8	2.8	2.0	1.2	0.0	0.0	0.4	0.0	0.0	0.7	0.4	0.8	0.8	0.0	0.0	0.4	1.6	0.9	1.7	0.5	0.5	0.0	2.4	0.0	0.0	0.0
CAF	-0.4	0.0	-2.5	-2.0	-1.2	0.0	0.3	-0.4	0.3	1.2	-0.7	-0.4	-0.2	-0.8	0.0	1.6	0.8	2.7	19.1	-0.1	-0.2	18.5	0.5	27.35	0.0	0.0	15.4
UMB	-0.4	0.0	0.4	-0.4	-1.2	0.4	0.0	-0.4	0.0	0.0	-0.7	-0.4	-0.8	-0.6	0.0	0.0	-0.1	-1.6	16.0	-1.1	-0.5	16.5	2.0	22.5	0.0	1.2	16.5
TPH	-0.4	0.0	-2.8	-2.0	-1.2	0.0	1.2	-0.4	0.0	0.0	-0.7	0.4	1.6	-0.8	0.0	0.0	-0.4	-1.6	20.3	0.7	-0.5	24.3	5.2	25.9	0.0	6.4	21.0
DEN	-0.4	0.0	-2.8	-2.0	-1.2	0.0	0.0	-0.4	0.0	0.0	-0.7	-0.4	-0.8	-0.8	0.0	0.0	-0.4	-1.6	-0.9	-1.7	-0.5	-0.5	0.0	8.0	0.0	0.0	0.0
BER	1.6	0.0	-2.4	-1.2	-0.4	0.8	2.0	-0.4	0.4	16	18.4	12.6	18.2	14.2	21.8	17.3	26.2	8.8	10.9	8.5	-0.3	20.3	8.2	24.8	0.0	2.4	9.2
LOB	2.4	0.0	-2.8	-2.0	-1.2	0.0	0.0	-0.4	0.0	12.7	15.5	8.0	7.6	8.1	10	15.3	22.0	11.1	14.7	-1.7	-0.5	16.3	0.0	18.5	0.3	0.0	15.3
SPS	-0.4	0.0	-2.8	-2.0	-1.2	0.0	0.0	-0.4	0.0	22.4	15.7	14.8	14.0	8.0	14	14.8	13.9	13.2	25.1	-1.7	-0.5	28.3	0.0	25.6	0.0	0.0	22.3
ESC	0.9	0.3	0.4	7.2	2.0	0.7	5.0	6.6	5.0	26.0	21.3	26.6	26.2	29.2	25	17.0	16.0	15.4	46.9	69.3	0.1	41.5	70.5	32.6	0.9	55	44.0
SAP	-0.4	0.0	-2.8	-2.0	-1.2	0.0	0.0	-0.4	0.0	0.0	-0.7	-0.4	-0.8	-0.8	0.0	0.0	-0.4	-1.6	14.8	23.1	-0.5	12.3	21.8	40.0	0.3	27.7	7.6
AZA	-0.4	0.0	-2.8	-2.0	-1.2	0.0	0.0	-0.4	0.0	0.0	-0.7	-0.4	-0.8	-0.8	0.0	0.0	-0.4	-1.6	12.4	-0.7	-0.5	5.5	0.9	0.4	0.0	0.8	4.0
COU	-0.4	0.0	-2.8	-2.0	-1.2	0.0	0.0	-0.4	0.0	0.0	-0.7	-0.4	-0.8	-0.8	0.0	0.0	-0.4	-1.6	40.4	-1.7	-0.5	36.3	0.0	33.9	0.0	0.0	32.0
SOA	-0.4	0.0	-2.8	-2.0	-1.2	0.0	0.0	-0.4	0.0	0.0	-0.7	-0.4	-0.8	-0.8	0.0	0.0	-0.4	-1.6	16.6	0.7	-0.5	15.7	0.8	16.0	0.0	3.3	14.0
DEET	-0.4	0.0	-2.8	-1.6	0.4	0.4	0.8	-0.4	0.0	0.0	-0.7	-0.4	-0.8	-0.8	0.0	0.0	-0.4	-1.6	31.8	-1.3	-0.5	31.8	0.9	17.6	0.0	1.6	23.7
STR	-0.4	0.0	-2.8	-2.0	-1.2	0.0	0.0	-0.4	0.0	0.0	-0.7	-0.4	-0.8	-0.8	0.0	0.0	-0.4	-1.6	-0.9	5.3	-0.5	-0.5	4.7	-2.4	0.0	4.4	0.0
GOS	-0.4	0.0	-2.8	-2.0	-1.2	0.0	0.0	-0.4	0.0	1.2	-0.7	-0.4	-0.8	-0.8	0.0	0.0	-0.4	-1.6	2.1	-1.7	-0.5	4.8	1.0	7.4	0.0	0.0	8.7
ARI	-0.4	0.0	-2.4	-1.2	-1.2	0.0	0.4	-0.4	0.8	0.0	-0.7	-0.4	-0.8	0.0	0.8	0.2	-0.4	-0.4	22.4	-1.7	-0.5	15.9	0.0	31.4	0.0	0.0	21

D. melanogaster

	L1	L2	L3	L4	L5	L6	L7	L8	L9	I0	I1	I2	I3	I4	I5	I6	I7	I8	I9	I10	S0	S1	S2	S3	S4	S5	S6	S7	S8	S9	S10
TCC	7.0	4.7	7.6	7.0	5.9	7.5	9.8	11.8	7.2	4.4	0.5	0.0	0.0	0.0	0.4	1.3	0.2	2.4	2.6	1.7	0.9	3.6	1.6	0.4	0.2	0.5	5.4	1.0	0.0	0.2	2.1
CAF	-4.0	-4.2	-5.3	-3.8	-1.6	-3.9	-1.5	-1.0	-5.2	4.2	0.7	0.0	1.7	0.5	6.2	-1.2	17.4	37.2	39.2	42.7	23.6	24.9	30.0	51.8	2.5	60.3	27.6	28.9	1.5	56.7	26.3
UMB	-1.6	0.7	1.3	-3.2	-1.9	-4.3	-5.8	-3.8	0.6	2.0	-0.3	0.0	0.7	0.0	1.4	2.5	13.8	38.6	35.8	35.8	13.6	18.8	21.5	46.8	-0.2	52.7	25.1	37.3	0.0	46.4	24.9
TPH	-1.9	-1.9	-4.1	-4.5	-2.1	-0.2	-9.5	-0.6	0.6	6.7	-0.5	2.7	0.0	0.3	-0.2	-1.2	15.6	43.7	40.0	41.1	11.9	9.4	18.8	44.7	-0.2	49.8	19.8	22.0	1.7	46.7	17.0
DEN	-6.5	-4.7	-6.5	-5.0	5.6	1.7	-8.0	-5.6	-4.9	21.5	19.2	19.7	23.9	21.6	21.8	17.9	16.3	1.1	-1.3	-0.6	28.3	17.2	35.2	32.2	1.1	43.6	20	30.4	0.0	37.1	18.4
BER	-3.4	-1.5	-5.8	-3.9	-3.0	-4.3	-7.3	-5.2	-4.5	15.7	34.2	29.5	31.6	34.1	29.8	23.5	18.1	8.0	1.1	6.9	23.4	17.0	27.1	10.0	-0.2	14.8	27.5	34.2	0.0	12.7	30.2
LOB	1.1	1.2	0.8	1.4	0.9	1.5	-1.6	-4.8	0.4	19.6	26.0	23.1	31.8	30.9	32.9	23.2	23.8	2.3	0.0	3.6	20.9	17.7	25.1	20.7	0.0	17.6	20.8	26.3	0.4	14.3	29.6
SPS	-3.6	-2.9	-2.0	-5.8	-3.6	-5.7	-7.6	-10.2	-5.8	9.1	8.1	10.5	11.3	18.6	15.9	9.4	12.2	-0.4	-2.2	-0.1	12.1	15.2	21.2	40.6	-0.2	36.8	16.7	33.9	0.0	35.4	15.2
ESC	-1.5	-2.3	0.8	0.2	-1.3	-2.5	0.1	-0.6	2.2	6.9	7.3	13.8	22	29.5	17.3	11.7	9.5	9.1	4.5	4.2	1.1	5.6	2.8	25.8	0.0	25.5	1.7	1.6	0.0	33.5	2.5
SAP	-3.2	3.3	-3.9	-1.5	-0.8	-3.7	-0.9	-4.4	-3.6	1.4	0.5	1.0	2.2	0.5	-0.1	2.3	6.0	15.4	7.6	17.1	16.6	15.4	14.6	38.3	1.0	51.3	20.2	36.8	0.0	50.2	11.5
AZA	1.4	-2.3	-2.3	-1.7	-3.2	-3.4	-1.5	-2.9	-0.6	-0.7	-0.5	0.0	0.0	0.0	1.4	-1.1	2.4	-1.2	-2.6	0.1	14.4	13.3	32.4	36.8	-0.2	39.8	35.2	29.3	0.0	40.8	27.8
COU	-3.2	2.7	-2.6	2.0	3.3	-1.3	-7.6	-6.8	-1.8	1.8	-0.5	5.2	0.0	0.2	0.0	-1.3	2.5	7.0	7.1	10.8	11.1	7.7	9.6	23.4	-0.2	30.9	9.2	17.6	0.0	35.7	9.1
SOA	-1.4	-0.9	-2.0	-3.7	-1.0	0.1	-9.2	-1.5	0.1	-3.5	-0.3	0.2	0.0	0.0	2.3	0.5	1.0	2.3	3.7	-0.2	18.5	10.2	19.2	21.9	-0.2	28.9	29.4	22.5	0.0	33.8	10.2
DEET	-1.9	-2.7	-5.1	-3.0	-3.3	-1.9	-5.0	-3.2	0.1	0.0	-0.5	0.0	0.0	1.5	-0.4	-1.3	-0.2	1.2	-1.6	-0.2	7.5	4.6	12.9	16.0	0.0	15.1	14.8	13.8	0.3	15.9	10.0
STR	-5.2	-1.5	-5.4	-4.6	-2.7	-4.7	-6.6	-8.4	-1.4	-3.6	-0.5	0.0	0.0	1.4	-0.4	-1.3	1.8	-0.4	0.2	0.7	6.5	4.4	8.6	22.8	-0.2	19.9	6.3	10.0	0.0	21.4	0.3
GOS	-5.9	-3.1	-6.3	-6.2	-5.5	-6.2	-4.0	-6.5	-5.6	-3.2	-0.5	0.0	0.0	0.0	-0.4	-1.3	3.0	-1.2	-0.4	3.8	4.7	4.6	5.2	8.8	-0.2	5.3	2.6	10.3	0.0	10.2	8.1
ARI	-5.2	-3.5	-5.6	-5.2	-3.9	-2.6	-2.0	-10.7	-3.6	-3.9	-0.5	0.0	0.2	0.0	-0.4	-1.3	0.4	-0.8	-1.0	-1.5	1.5	3.4	5.7	13.0	-0.2	5.7	11.1	6.4	0.0	5.8	6.0

B

D. suzukii

	L1	L2	L3	L4	L5	L6	L7	L8	L9	I0	I1	I2	I3	I4	I5	I6	I7	I8	S1	S2	S3	S4	S5	S6	S7	S8	S9
TCC	0.0	0.0	0.0	0.0	0.0	0.0	0.0	0.0	0.0	0.0	0.0	0.0	0.0	0.0	0.0	0.0	0.0	0.0	0.8	1.3	0.3	0	0.7	1.6	0.6	1.0	0.0
CAF	0.0	0.0	0.0	0.0	0.0	0.7	0.0	0.0	0.0	1.8	0.5	1.6	1.5	1.1	0.9	1.6	1.5	0.5	0.9	1.5	1.2	1.9	1.7	1.4	1.6	2.6	1.4
UMB	0.8	0.0	0.8	0.7	0.0	0.0	0.0	0.4	0.4	1.7	0.8	2.2	1.4	2.0	1.9	1.5	0.6	0.7	2.4	1.7	0.3	1.6	4.2	5.4	1.2	2.1	1.9
TPH	0.3	0.0	0.5	0.0	0.0	0.0	0.0	0.0	0.0	0.9	0.7	1.2	1.8	0.8	1.2	1.2	0.9	0.2	2.8	0.4	0.4	1.4	0.5	1.0	0.3	0.6	1.2
DEN	0.0	0.4	0.3	0.0	0.0	0.0	0.0	0.0	0.0	0.0	0.4	1.1	0.7	0.3	1.0	0.8	1.2	1.0	1.5	0.0	0.0	1.1	0.0	1.8	0.0	0.0	2.7
BER	0.0	0.3	0.0	0.0	0.4	0.4	0.0	0.0	0.8	1.6	0.9	1.8	1.7	0.9	2.1	1.5	3.0	3.3	1.6	1.0	1.8	1.5	0.5	2.1	0.3	0.6	2.0
LOB	0.5	0.0	0.0	0.0	0.0	0.0	0.0	0.0	0.0	1.7	2.2	1.6	1.7	2.6	1.9	2.6	2.6	1.1	1.4	0.0	0.0	1.4	0.3	3.2	0.3	0.9	1.9
SPS	0.0	0.0	0.0	0.0	0.0	0.0	0.0	0.0	0.0	2.9	2.0	2.2	1.3	1.8	2.2	1.4	2.7	1.8	2.1	0.5	0.3	0.4	0.0	2.0	0.0	0.0	1.0
ESC	0.0	0.0	0.0	0.0	0.3	0.0	0.0	0.0	0.0	1.5	2.6	1.6	1.3	2.5	1.6	0.7	1.4	0.6	2.7	1.4	0.7	1.6	1.3	2.5	0.7	1.2	7.0
SAP	0.5	0.0	0.0	0.9	0.0	0.0	0.4	1.2	0.4	0.7	0.0	0.8	0.0	0.8	0.4	1.3	0.4	0.0	1.1	0.5	0.0	1.5	1.9	2.6	0.3	2.3	0.4
AZA	0.4	0.0	0.0	0.4	0.4	0.5	0.0	0.0	0.7	0.0	0.4	0.6	1.5	1.0	0.0	0.7	0.4	0.0	2.9	2.5	0.0	0.8	0.8	2.7	0.6	1.3	1.0
COU	0.0	0.0	0.0	0.0	0.0	0.0	0.0	0.0	0.0	0.5	1.9	0.6	1.2	0.8	0.8	0.4	1.0	0.8	3.0	0.9	1.6	1.5	0.4	2.6	0.0	1.6	1.0
SOA	0.0	0.0	0.0	0.0	0.0	0.0	0.0	0.0	0.0	0.4	0.0	0.8	1.8	0.0	0.0	0.0	0.0	0.0	2.9	0.0	0.0	0.9	0.0	4.0	0.0	0.0	1.3
DEET	0.0	0.0	0.0	0.0	0.0	0.0	0.0	0.0	0.0	0.0	0.0	0.0	0.3	0.0	0.4	0.0	0.4	0.0	0.7	0.4	0.0	1.4	0.0	1.0	0.0	0.0	0.7
STR	0.0	0.0	0.0	0.0	0.0	0.0	0.0	0.0	0.4	0.0	0.0	0.0	0.4	0.0	0.0	0.0	0.0	0.4	4.4	0.0	0.0	0.8	0.4	6.2	0.0	0.0	1.7
GOS	0.0	0.0	0.0	0.0	0.0	0.0	0.0	0.0	0.0	0.0	0.0	0.3	0.0	0.0	0.0	0.0	0.0	0.0	0.7	3.5	0.3	0.3	4.4	4.0	0.0	3.0	0.5
ARI	0.0	0.0	0.0	0.0	0.0	0.0	0.0	0.0	0.0	0.5	0.4	0.5	0.0	0.4	0.4	0.0	0.0	0.4	3.3	0.9	0.7	1.9	3.3	3.7	0.8	4.0	3.1

D. biarmipes

	L1	L2	L3	L4	L5	L6	L7	L8	L9	I0	I1	I2	I3	I4	I5	I6	I7	I8	S1	S2	S3	S4	S5	S6	S7	S8	S9
TCC	0.4	0.8	0.5	0.0	0.8	0.0	0.0	0.4	0.0	0.0	0.7	0.4	0.5	0.8	0.0	0.0	0.4	0.4	0.9	0.0	0.0	0.4	0.0	0.4	0.0	0.0	0.0
CAF	0.0	0.0	0.3	0.0	0.0	0.0	0.3	0.0	0.3	0.8	0.0	0.0	0.4	0.0	0.0	1.0	0.6	1.5	1.8	0.4	0.3	2.1	0.4	1.8	0.0	0.0	0.6
UMB	0.0	0.0	0.0	0.0	0.0	0.4	0.0	0.0	0.0	0.0	0.0	0.0	0.0	0.2	0.0	0.0	0.3	0.0	0.8	0.4	0.0	2.3	0.6	3.3	0.0	1.2	1.5
TPH	0.0	0.0	0.0	0.0	0.0	0.0	1.2	0.0	0.0	0.0	0.0	0.5	0.4	0.0	0.0	0.0	0.0	0.0	2.9	1.0	0.0	2.9	2.4	4.7	0.0	2.4	2.4
DEN	0.0	0.0	0.0	0.0	0.0	0.0	0.0	0.0	0.0	0.0	0.0	0.0	0.0	0.0	0.0	0.0	0.0	0.0	0.0	0.0	0.0	0.0	0.0	1.3	0.0	0.0	0.0
BER	0.5	0.0	0.4	0.8	0.5	0.5	0.5	0.0	0.4	1.6	3.4	1.4	4.4	1.6	2.4	3.8	2.9	1.3	1.2	1.6	0.2	1.5	0.5	2.0	0.0	1.7	1.2
LOB	0.5	0.0	0.0	0.0	0.0	0.0	0.0	0.0	0.0	2.0	2.0	2.0	1.3	1.1	1.5	1.0	1.3	0.8	1.8	0.0	0.0	0.5	0.0	1.3	0.3	0.0	4.0
SPS	0.0	0.0	0.0	0.0	0.0	0.0	0.0	0.0	0.0	2.6	0.4	2.3	1.9	0.8	0.9	1.2	1.5	1.9	1.5	0.0	0.0	1.6	0.0	2.4	0.0	0.0	1.6
ESC	0.7	0.3	2.7	1.5	1.6	0.7	0.9	3.4	1.7	2.0	1.4	3.8	1.2	5.0	1.4	5.2	4.0	0.9	2.7	4.4	0.3	3.6	3.2	6.0	0.9	6.8	4.0
SAP	0.0	0.0	0.0	0.0	0.0	0.0	0.0	0.0	0.0	0.0	0.0	0.0	0.0	0.0	0.0	0.0	0.0	0.0	1.1	6.5	0.0	1.4	3.1	5.0	0.0	5.4	2.2
AZA	0.0	0.0	0.0	0.0	0.0	0.0	0.0	0.0	0.0	0.0	0.0	0.0	0.0	0.0	0.0	0.0	0.0	0.0	2.2	0.4	0.0	1.4	0.5	0.5	0.0	0.5	0.6
COU	0.0	0.0	0.0	0.0	0.0	0.0	0.0	0.0	0.0	0.0	0.0	0.0	0.0	0.0	0.0	0.0	0.0	0.0	2.5	0.0	0.0	1.9	0.0	0.8	0.0	0.0	4.2
SOA	0.0	0.0	0.0	0.0	0.0	0.0	0.0	0.0	0.0	0.0	0.0	0.0	0.0	0.0.	0.0	0.0	0.0	0.0	2.1	0.9	0.0	0.8	0.3	2.9	0.0	1.4	1.0
DEET	0.0	0.0	0.0	0.4	0.7	0.4	0.5	0.0	0.0	0.0	0.0	0.0	0.0	0.0	0.0	0.0	0.0	0.0	4.3	0.4	0.0	2.6	0.6	3.6	0.0	0.7	2.0
STR	0.0	0.0	0.0	0.0	0.0	0.0	0.0	0.0	0.0	0.0	0.0	0.0	0.0	0.0	0.0	0.0	0.0	0.0	0.0	1.4	0.0	0.0	1.0	0.0	0.0	1.2	0.0
GOS	0.0	0.0	0.0	0.0	0.0	0.0	0.0	0.0	0.0	0.5	0.0	0.0	0.0.	0.0	0.0	0.0	0.0	0.0	0.5	0.0	0.0	1.1	0.7	2.7	0.0	0.0	1.7
ARI	0.0	0.0	0.4	0.5	0.0	0.0	0.4	0.0	0.5	0.0	0.0	0.0	0.0	0.8	0.5	0.2	0.0	0.8	1.8	0.0	0.0	2.3	0.0	0.8	0.0	0.0	2.0

D. melanogaster

	L1	L2	L3	L4	L5	L6	L7	L8	L9	I0	I1	I2	I3	I4	I5	I6	I7	I8	I9	I10	S0	S1	S2	S3	S4	S5	S6	S7	S8	S9	S10
TCC	1.1	1.1	1.2	1.6	1.4	1.2	1.6	1.5	1.0	0.9	0.5	0.0	0.0	0.0	0.3	0.8	0.1	0.9	0.8	0.7	0.4	2.2	0.9	0.3	0.2	0.3	1.2	0.5	0.0	0.1	0.9
CAF	2.6	0.4	0.8	1.3	2.5	1.4	2.3	3.3	0.8	2.3	1.2	0.0	1.2	0.4	3.4	0.1	3.3	3.1	3.2	2.8	3.6	3.8	3.4	3.6	2.6	3.3	2.9	3.0	1.5	3.9	2.6
UMB	1.9	2.2	3.1	1.8	1.7	1.1	1.6	3.6	2.7	3.7	0.2	0.0	0.6	0.0	1.6	2.8	5.0	6.4	7.4	7.4	3.4	5.2	3.3	4.3	0.0	4.8	2.8	3.6	0.0	3.3	4.6
TPH	1.9	1.6	1.9	1.1	1.6	2.6	2.7	2.7	2.6	2.8	0.0	2.4	0.0	0.3	0.2	0.1	5.1	5.7	4.0	3.3	3.0	4.4	4.1	4.8	0.0	5.9	3.2	3.0	1.7	4.2	3.5
DEN	0.3	0.0	0.4	1.4	6.8	6.7	0.6	2.4	1.1	4.0	6.6	4.0	3.9	3.5	3.2	2.7	2.9	1.4	0.6	0.5	2.9	3.1	3.5	4.5	1.3	3.0	1.8	3.0	0.0	3.9	2.7
BER	1.3	1.4	0.9	1.2	1.4	1.4	0.9	1.8	0.8	2.7	3.7	3.0	2.7	3.3	3.6	2.7	2.4	2.1	1.5	1.9	3.4	2.9	2.1	3.5	0.0	3.0	1.8	2.3	0.0	4.4	2.1
LOB	3.0	1.7	2.6	3.9	1.8	2.3	2.1	2.2	2.9	6.0	6.5	3.2	5.0	4.8	4.8	6.3	4.9	2.8	1.6	2.2	3.7	3.0	5.6	5.0	0.2	3.5	2.7	3.0	0.4	2.5	3.0
SPS	1.2	1.0	2.9	1.0	1.2	1.4	1.4	1.0	0.5	3.5	3.2	3.3	3.6	4.3	3.9	3.8	3.6	1.3	0.3	0.7	4.0	2.2	3.0	4.5	0.0	5.5	2.8	2.8	0.0	5.7	2.9
ESC	2.1	1.1	2.0	2.6	1.9	2.1	3.1	4.1	2.3	4.2	3.9	5.2	6.7	6.5	5.6	4.2	4.0	4.7	2.5	2.4	1.2	3.2	2.3	3.8	0.2	5.6	1.8	1.3	0.0	5.8	1.5
SAP	1.4	4.0	1.1	1.5	3.5	1.2	2.6	1.9	1.1	1.9	1.0	1.0	1.7	0.5	0.3	2.3	4.5	4.1	1.9	6.0	3.9	4.1	5.0	3.5	0.8	5.0	4.7	7.9	0.0	3.6	4.9
AZA	2.9	0.9	1.7	1.4	1.4	1.3	2.3	2.4	2.6	1.8	0.0	0.0	0.0	0.0	1.2	0.2	2.4	1.2	0.0	0.9	3.0	3.6	3.1	4.0	0.0	3.4	3.1	2.8	0.0	4.8	2.7
COU	1.4	2.3	1.9	3.7	3.6	2.4	0.8	1.9	3.5	6.0	0.0	5.2	0.0	0.2	0.4	0.0	2.7	3.8	4.3	5.2	4.4	4.4	3.7	6.5	0.0	7.1	4.8	5.3	0.0	4.3	4.6
SOA	2.2	3.0	2.4	1.2	2.9	1.9	2.7	2.6	3.1	0.6	0.2	0.2	0.0	0.0	1.9	1.3	0.6	3.6	3.3	1.1	5.0	3.3	4.7	4.5	0.0	4.6	3.4	4.1	0.0	5.1	5.1
DEET	1.6	1.0	1.3	2.2	0.9	1.5	2.5	3.2	2.3	1.7	0.0	0.0	0.0	1.5	0.0	0.0	0.0	3.0	0.8	0.9	2.6	2.5	2.8	2.2	0.2	3.5	2.6	3.8	0.3	3.3	1.5
STR	1.2	1.6	1.6	1.5	2.0	2.4	1.2	2.0	1.8	0.6	0.0	0.0	0.0	1.4	0.0	0.0	2.0	1.8	1.3	1.1	2.3	1.7	3.1	3.9	0.0	3.3	2.1	2.0	0.0	5.0	0.8
GOS	0.6	1.0	0.7	0.6	0.4	0.6	1.9	1.3	0.6	0.7	0.0	0.0	0.0	0.0	0.0	0.0	2.6	0.8	2.2	2.5	2.3	3.6	2.2	2.4	0.0	2.1	1.8	5.5	0.0	3.8	2.4
ARI	0.9	1.2	0.8	1.1	1.0	1.8	1.7	3.2	2.3	0.5	0.0	0.0	0.2	0.0	0.0	0.0	0.6	1.4	0.9	0.2	1.0	2.6	2.4	4.4	0.0	2.1	2.6	2.3	0.0	2.5	1.9
