# Supplementary material for: Evolutionary shifts in taste coding in the fruit pest Drosophila suzukii
Source: eLife. 2021 Feb 22;10:e64317. doi: 10.7554/eLife.64317 (PMC7899650; doi:10.7554/eLife.64317)
Supplement: Figure 7—source data 1. — Values represent the mean ± SEM responses of spikes/s. n = 5–17. Responses to the diluent control, tricholine citrate (TCC), were subtracted. [file elife-64317-fig7-data1.pdf]

Table. S2

D. suzukii

	f5v	f5S	f5b	f5a	f4c	f4s	f4b
TCC	0.0 ± 0.0	23.4 ± 2.8	0.0 ± 0.0	0.0 ± 0.0	0.0 ± 0.0	0.0 ± 0.0	0.0 ± 0.0
CAF	0.0 ± 0.0	21.9 ± 3.3	2.4 ± 1.5	0.0 ± 0.0	0.0 ± 0.0	0.0 ± 0.0	0.0 ± 0.0
UMB	0.0 ± 0.0	1.4 ± 1.6	0.0 ± 0.0	0.0 ± 0.0	0.0 ± 0.0	0.0 ± 0.0	0.0 ± 0.0
TPH	0.0 ± 0.0	-2.6 ± 4.4	0.0 ± 0.0	0.0 ± 0.0	0.0 ± 0.0	0.0 ± 0.0	0.0 ± 0.0
DEN	0.0 ± 0.0	15.5 ± 3.8	34.3 ± 3.6	0.0 ± 0.0	26.8 ± 3.6	34.5 ± 2.1	0.0 ± 0.0
BER	0.0 ± 0.0	11.8 ± 1.4	0.0 ± 0.0	0.0 ± 0.0	0.0 ± 0.0	0.0 ± 0.0	0.0 ± 0.0
LOB	0.0 ± 0.0	13.3 ± 3.1	20.4 ± 2.2	0.0 ± 0.0	26.2 ± 2.4	21.6 ± 1.7	0.0 ± 0.0
SPS	0.0 ± 0.0	30.6 ± 3.5	11.0 ± 1.0	0.0 ± 0.0	9.2 ± 1.4	12.0 ± 2.0	0.0 ± 0.0
ESC	0.0 ± 0.0	-1.4 ± 2.6	0.0 ± 0.0	0.0 ± 0.0	0.0 ± 0.0	0.0 ± 0.0	0.0 ± 0.0
SAP	0.0 ± 0.0	-11.0 ± 1.6	0.0 ± 0.0	0.0 ± 0.0	0.0 ± 0.0	0.0 ± 0.0	0.0 ± 0.0
AZA	0.0 ± 0.0	-1.0 ± 2.4	0.0 ± 0.0	0.0 ± 0.0	2.4 ± 1.5	0.0 ± 0.0	0.0 ± 0.0
COU	0.0 ± 0.0	-4.6 ± 2.3	0.0 ± 0.0	0.0 ± 0.0	0.0 ± 0.0	0.6 ± 0.4	0.0 ± 0.0
SOA	0.0 ± 0.0	-4.6 ± 1.0	0.0 ± 0.0	0.0 ± 0.0	0.0 ± 0.0	0.0 ± 0.0	0.0 ± 0.0
DEET	0.0 ± 0.0	-5.8 ± 2.3	0.0 ± 0.0	0.0 ± 0.0	0.0 ± 0.0	0.8 ± 0.5	0.0 ± 0.0
STR	0.0 ± 0.0	14.5 ± 1.1	17.6 ± 1.2	0.0 ± 0.0	20.8 ± 0.8	16.8 ± 1.9	0.0 ± 0.0
GOS	0.0 ± 0.0	-0.6 ± 2.4	0.0 ± 0.0	0.0 ± 0.0	0.0 ± 0.0	0.0 ± 0.0	0.0 ± 0.0
ARI	0.0 ± 0.0	11.9 ± 1.0	0.0 ± 0.0	0.0 ± 0.0	0.0 ± 0.0	0.0 ± 0.0	0.0 ± 0.0

D. biarmipes

	F5v	F5s	F5b	F5a	F4c	F4s	F4b
TCC	0.0 ± 0.0	19.0 ± 2.1	0.0 ± 0.0	0.0 ± 0.0	0.0 ± 0.0	0.0 ± 0.0	0.0 ± 0.0
CAF	0.0 ± 0.0	0.2 ± 1.7	2.4 ± 1.5	0.0 ± 0.0	0.4 ± 0.4	2.0 ± 1.1	0.0 ± 0.0
UMB	14.0 ± 1.8	6.2 ± 2.3	0.0 ± 0.0	0.0 ± 0.0	0.0 ± 0.0	0.0 ± 0.0	0.0 ± 0.0
TPH	0.0 ± 0.0	-2.2 ± 3.4	0.0 ± 0.0	0.0 ± 0.0	0.0 ± 0.0	0.0 ± 0.0	0.0 ± 0.0
DEN	0.4 ± 0.4	1.4 ± 1.7	21.6 ± 4.1	0.0 ± 0.0	17.2 ± 1.6	17.2 ± 2.6	0.0 ± 0.0
BER	0.0 ± 0.0	10.1 ± 1.9	3.2 ± 1.4	0.0 ± 0.0	0.0 ± 0.0	0.0 ± 0.0	0.0 ± 0.0
LOB	0.0 ± 0.0	-0.2 ± 2.1	34.4 ± 4.0	0.0 ± 0.0	12.4 ± 1.5	33.3 ± 2.9	0.0 ± 0.0
SPS	14.2 ± 1.1	18.3 ± 1.5	13.2 ± 0.6	0.0 ± 0.0	0.9 ± 0.4	10.8 ± 1.4	0.0 ± 0.0
ESC	0.0 ± 0.0	2.3 ± 2.8	0.0 ± 0.0	0.0 ± 0.0	0.0 ± 0.0	0.0 ± 0.0	0.0 ± 0.0
SAP	0.0 ± 0.0	3.0 ± 2.8	0.0 ± 0.0	0.0 ± 0.0	0.0 ± 0.0	0.0 ± 0.0	0.0 ± 0.0
AZA	0.0 ± 0.0	-1.0 ± 1.7	0.0 ± 0.0	0.0 ± 0.0	0.0 ± 0.0	0.4 ± 0.0	0.0 ± 0.0
COU	0.8 ± 0.5	-3.0 ± 1.2	12.0 ± 1.0	0.0 ± 0.0	8.8 ± 0.5	14.4 ± 1.5	0.0 ± 0.0
SOA	0.0 ± 0.0	-1.8 ± 1.0	0.0 ± 0.0	0.0 ± 0.0	0.0 ± 0.0	0.0 ± 0.0	0.0 ± 0.0
DEET	0.0 ± 0.0	3.0 ± 3.0	17.5 ± 2.2	0.0 ± 0.0	15.6 ± 1.7	15.0 ± 1.6	0.0 ± 0.0
STR	18.4 ± 1.5	-7.8 ± 0.8	15.7 ± 1.6	0.0 ± 0.0	15.6 ± 1.2	9.2 ± 0.8	0.0 ± 0.0
GOS	0.0 ± 0.0	-5.4 ± 1.3	0.0 ± 0.0	0.0 ± 0.0	0.0 ± 0.0	0.0 ± 0.0	0.0 ± 0.0
ARI	17.2 ± 1.4	1.4 ± 1.5	0.0 ± 0.0	0.0 ± 0.0	0.0 ± 0.0	0.0 ± 0.0	0.0 ± 0.0

D. melanogaster

	F5v	F5s	F5b	F5a	F4c	F4s	F4b
TCC	0.0 ± 0.0	15.2 ± 1.0	0.0 ± 0.0	0.0 ± 0.0	0.0 ± 0.0	0.0 ± 0.0	0.0 ± 0.0
CAF	0.0 ± 0.0	10.0 ± 0.5	0.0 ± 0.0	0.0 ± 0.0	0.0 ± 0.0	0.0 ± 0.0	0.0 ± 0.0
UMB	0.0 ± 0.0	5.6 ± 0.0	0.0 ± 0.0	0.0 ± 0.0	0.0 ± 0.0	0.0 ± 0.0	0.0 ± 0.0
TPH	0.0 ± 0.0	1.4 ± 2.5	0.0 ± 0.0	0.0 ± 0.0	0.0 ± 0.0	0.0 ± 0.0	0.0 ± 0.0
DEN	0.0 ± 0.0	15.2 ± 0.7	34.8 ± 2.3	0.0 ± 0.0	28.8 ± 1.6	26.0 ± 2.3	0.0 ± 0.0
BER	0.0 ± 0.0	20.0 ± 2.7	0.0 ± 0.0	0.0 ± 0.0	0.0 ± 0.0	0.0 ± 0.0	0.0 ± 0.0
LOB	0.0 ± 0.0	9.8 ± 0.7	29.2 ± 2.4	0.0 ± 0.0	38.5 ± 3.3	39.2 ± 4.2	0.0 ± 0.0
SPS	0.0 ± 0.0	35.1 ± 1.3	30.0 ± 1.7	0.0 ± 0.0	28.3 ± 2.0	22.0 ± 1.8	0.0 ± 0.0
ESC	0.0 ± 0.0	1.8 ± 3.1	0.4 ± 0.4	0.0 ± 0.0	0.0 ± 0.0	0.0 ± 0.0	0.0 ± 0.0
SAP	0.0 ± 0.0	-4.4 ± 0.5	0.4 ± 0.4	0.0 ± 0.0	0.0 ± 0.0	0.0 ± 0.0	0.0 ± 0.0
AZA	0.0 ± 0.0	0.6 ± 1.5	0.8 ± 0.5	0.0 ± 0.0	4.4 ± 2.0	1.2 ± 1.2	0.0 ± 0.0
COU	0.0 ± 0.0	5.0 ± 2.0	16.0 ± 2.0	0.0 ± 0.0	18.4 ± 1.2	20.3 ± 2.4	0.0 ± 0.0
SOA	0.0 ± 0.0	3.2 ± 1.2	0.0 ± 0.0	0.0 ± 0.0	0.0 ± 0.0	0.0 ± 0.0	0.0 ± 0.0
DEET	0.0 ± 0.0	8.2 ± 4.8	24.0 ± 1.6	0.0 ± 0.0	16.4 ± 1.2	18.6 ± 2.1	0.0 ± 0.0
STR	0.0 ± 0.0	8.6 ± 1.7	15.6 ± 0.7	0.0 ± 0.0	15.3 ± 1.8	17.2 ± 3.0	0.0 ± 0.0
GOS	0.0 ± 0.0	-2.4 ± 1.2	0.0 ± 0.0	0.0 ± 0.0	0.0 ± 0.0	0.0 ± 0.0	0.0 ± 0.0
ARI	0.0 ± 0.0	3.4 ± 1.0	0.0 ± 0.0	0.0 ± 0.0	2.4 ± 0.4	0.0 ± 0.0	0.0 ± 0.0
